# Closer Affiliation between Medicine and Dentistry

**Published:** 1921-09

**Authors:** E. C. Kirk


### CLOSER AFFILIATION BETWEEN MEDICINE AND DENTISTRY

For several decades, in fact, almost from the beginning of dentistry as a specialty of medicine, dentists have endeavored to obtain proper recognition from the medical profession, but without avail, except in a few isolated cases. It is only within the past few years that there has occurred a change in the attitude of the medical profession, and that as a result of the awakening of the medical profession to the insidious nature of oral focal infections in their bearing upon systematic conditions and the consequent recognition of the necessity for a better knowledge of mouth conditions, or in lieu thereof a closer affiliation with the dental profession to the end that they may utilize the dentist's knowledge in combating some of the diseases which have been shown to come from oral focal infection.

The medical profession, since the very origin of dentistry as a separate specialty, has consistently assumed an attitude of aloofness toward dentistry, and has consistently refused to recognize dentistry as an important adjunct to the healing art. Indeed, it was this attitude on the part of medicine that was the impelling and compelling motive which prompted Harris and Hayden in 1839 to withdraw from the faculty of the Medical Department of the University of Maryland and establish a separate school for the education of dentists.

It has taken the medical profession several generations to see the mistake that was then made and it is only recently, and that by virtue of the enforced recognition of the importance of oral focal infection as a factor in the cause of many intractable diseases which they had been unable to account for otherwise, that many of the leading members of the medical profession have expressed their willingness, even readiness, to accord to dentistry its rightful place as a branch of the great healing art and have indicated quite clearly their desire to take the dentist into their councils in the hope that he may help to solve some of their many problems.

The most recent expression of this changed attitude on the part of medicine is voiced by the *Medical Record* in an editorial in that journal published in March, 1921, and we trust the editor is acting as the mouthpiece of the greater portion of the profession. In an appeal for closer affiliation between the two professions the editor says:

"This mistake of aloofness or assumed superiority on the part of the medical profession was made when dentistry began to acquire a standing as a branch of therapeusis, with the result that the two professions have long existed side by side, semi-antagonistic, and mutually ignorant of each other's teaching.

"It is only recently that the inconvenience of this duality in surgical and medical practice has begun to enter the minds of practitioners of both the professions, and in consequence there has been a mutual drawing together of the leaders in these two branches of the healing art. The recognition of root abscesses and Riggs' disease as foci of infection, bearing upon distant organs and even the brain, has served to show the mutual dependence of the two professions, but it was during the late war that the recognition of dentistry as a potential or actual branch of surgery was effected, this being forced upon the Army Medical Department by the brilliant reconstructive work of the members of the Dental Reserve Corps. There will still be accidents calling for skilled work of this nature, but it is along the line of preventive medicine and hygiene that the co-operation of dentist and physician will produce its greatest results. The public must be instructed in mouth hygiene, which is inseparable from general hygiene, and for this work reciprocal action of members of both professions on the platform and in the press is needed to feed the public appetite for knowledge of how to care for the body.

"In many other ways the dentist and the physician could work together to mutual advantage, and as a beginning, an 'exchange of pulpits' would greately promote the desired *rapprochement.* It is seldom that a dentist reads a paper before a medical society, and a physician speaking before a dental society is a still more rare object. This is not as it should be, for practitioners in either profession have much to learn from those in the other, and preventive medicine as well as therapeutics would profit greatly by this mutual instruction."

Some years ago we expressed the hope that one of the beneficial results of the World War would be to draw the two professions closer together, and we felt confident that in this closer relationship, brought about by the necessity of the dentist's skill as a means of maintaining the highest health efficiency of the soldiers, the dentist would prove his worth, and it seems that we are fully justified in such confidence. The Dental Corps did all and more than was expected of them under most trying circumstances, and we feel still further confident that the dentist can prove his specialty to be of invaluable aid in maintaining the health efficiency of the community in co-operation with the medical man if he be given the opportunity.

It seems that this recognition for which we have clamored for years is gradually coming, as medical men generally are in a more receptive attitude toward receiving the advice of dentists. Not all, however, are ready to take his advice, as there are still many who arrogate to themselves the authority to speak with finality on the course which shall be pursued in dealing with mouth conditions which are suspected of relationship to systemic disease, and as a result we quite frequently hear of patients being referred to dentists with instructions to remove certain teeth, and in some instances all teeth, without asking the dentist's advice as to whether or not the teeth are or may be made useful organs of mastication.

Orders given by physicians for the extraction of teeth, in relation to which a diagnosis of focal infection has been made, are invariably issued in total ignorance of the resources of modern dentistry available for the elimination of dental focal infections by means other than extraction. A fundamental fact constantly overlooked or ignored by physicians is that it is not the tooth as such that is the source of disease, but the pathogenic micro-organisms that have found a habitat in relation to the tooth that constitutes the *fons et origo* of disease causation.

It has been clearly demonstrated today that dental therapeusis is competent to deal successfully with these foci of infection without removal of the tooth in a majority of cases, so that practically, as well as theoretically, there is no more justification for the removal of a tooth, the seat of focal infection, than there is for the amputation of a finger which is the seat of a whitlow.

The right of judgment is dependent upon knowledge of the data presented by the case and the means available for relief of the conditions present. This is specialized knowledge that the physician does not possess, and to order the extraction of teeth in such circumstances is an arbitrary assumption of authority, which at present he has no moral right to claim. The dentist should in such cases be the final arbiter.

One of the tasks, and we use the word advisedly, of the dentist will be to teach his medical brother the fact that the mouth and teeth serve an important function as an essential factor of the digestive process, and that the teeth are not to be indiscriminately sacrificed simply as a precautionary measure against possible systemic complications or merely as a step in the process of diagnosis by exclusion.

The greatest help that dentistry can give to medicine in its efforts to maintain health in the community, will be in the direction of preventive medicine, since it has been indisputably proved that dental diseases are so prevalent and have such potentiality for evil in their relation to general bodily health. Neither medicine nor dentistry can hope to attain the goal implied in what we term *preventive medicine* without the aid of the other, and we venture the prophecy that this goal will be more quickly and effectively attained if the efforts of the two professions can be brought together as one strong body working in collaboration in the field of preventive hygiene and therapeutics.

Now that this opportunity of co-operation is seemingly near at hand, it behooves the dental profession to gracefully accept it and above all to place a just estimate upon the responsibilities it has thus brought upon itself to the end that they may be discharged with only the one great *motif* of service to humanity in view.—Dr. E. C. Kirk, in *Dental Cosmos,* July, 1921.

